# Electrosprayed Chitin Nanofibril/Electrospun Polyhydroxyalkanoate Fiber Mesh as Functional Nonwoven for Skin Application

**DOI:** 10.3390/jfb11030062

**Published:** 2020-09-03

**Authors:** Bahareh Azimi, Lily Thomas, Alessandra Fusco, Ozlem Ipek Kalaoglu-Altan, Pooja Basnett, Patrizia Cinelli, Karen De Clerck, Ipsita Roy, Giovanna Donnarumma, Maria-Beatrice Coltelli, Serena Danti, Andrea Lazzeri

**Affiliations:** 1 Interuniversity National Consortiums of Materials Science and Technology (INSTM), 50121 Firenze FL, Italy; b.azimi@ing.unipi.it (B.A.); alessandra.fusco@unicampania.it (A.F.); patrizia.cinelli@unipi.it (P.C.); giovanna.donnarumma@unicampania.it (G.D.); maria.beatrice.coltelli@unipi.it (M.B.-C.); 2 Department of Civil and Industrial Engineering, University of Pisa, 56126 Pisa PI, Italy; andrea.lazzeri@unipi.it; 3 Schools of Biosciences, Cardiff University, Cardiff CF10 3AT, UK; thomaslm13@cardiff.ac.uk; 4 Department of Experimental Medicine, University of Campania “Luigi Vanvitelli”, 81100 Caserta CE, Italy; 5 Centre for Textile Science and Engineering, Department of Materials, Textiles and Chemical Engineering, 9000 Gent, Belgium; ozlemkalaoglu@gmail.com (O.I.K.-A.); karen.declerck@ugent.be (K.D.C.); 6 School of Life Sciences, College of Liberal Arts and Sciences, University of Westminster, London W1W 7BY, UK; p.basnett@westminster.ac.uk; 7 Department of Materials Science & Engineering, Kroto Research Institute, University of Sheffield, Sheffield S10 2TG, UK; i.roy@sheffield.ac.uk

**Keywords:** biopolymer, bio-based, surface modification, nanolignin, electrospinning, electrospray, anti-inflammatory

## Abstract

Polyhydroxyalkanoates (PHAs) are a family of bio-based polyesters that have found different biomedical applications. Chitin and lignin, byproducts of fishery and plant biomass, show antimicrobial and anti-inflammatory activity on the nanoscale. Due to their polarities, chitin nanofibril (CN) and nanolignin (NL) can be assembled into micro-complexes, which can be loaded with bioactive factors, such as the glycyrrhetinic acid (GA) and CN-NL/GA (CLA) complexes, and can be used to decorate polymer surfaces. This study aims to develop completely bio-based and bioactive meshes intended for wound healing. Poly(3-hydroxybutyrate)/Poly(3-hydroxyoctanoate-co-3-hydroxydecanoate), P(3HB)/P(3HO-*co*-3HD) was used to produce films and fiber meshes, to be surface-modified via electrospraying of CN or CLA to reach a uniform distribution. P(3HB)/P(3HO-*co*-3HD) fibers with desirable size and morphology were successfully prepared and functionalized with CN and CLA using electrospinning and tested in vitro with human keratinocytes. The presence of CN and CLA improved the indirect antimicrobial and anti-inflammatory activity of the electrospun fiber meshes by downregulating the expression of the most important pro-inflammatory cytokines and upregulating human defensin 2 expression. This natural and eco-sustainable mesh is promising in wound healing applications.

## 1. Introduction

Polyhydroxyalkonates (PHAs) have recently attracted wide interest in research and product development, as the biomedical market has started to look for better sustainable alternatives to typical petroleum-based products [1]. In nature, PHAs exist as intracellular energy storage and can be produced by various microorganisms. Therefore, these macromolecules demonstrate very good biocompatibility, which has been extensively studied in multiple cell lines, making them ideal polymers for biomedical applications to be produced inside fermenters [2]. Along with low toxicity towards mammalian tissues, PHAs may also show an antimicrobial and bactericidal effect, a key property than can be utilized in the development of wound dressings for tissue regeneration [3]. The immunomodulatory properties of these materials also make them particularly useful in the wound repair process. PHA/starch blend films also showed potential for innovative bio-based beauty masks, wearable after wetting, and releasing starch [4,5]. Different fermentation process conditions result in a variety of PHAs with different properties—for example, they can span from a rigid to an elastomeric behavior [6]. Polyhydroxybutyrate, P(3HB), one of the more studied PHAs holds promise with a lower inflammatory effect in vivo, due to lower acidity of the released hydroxy acids compared to other widely available bioplastics, such as polylactic acid (PLA) [1]. A downfall of P(3HB) is its tendency to low processability and brittleness [1], but through co-polymerization, these limitations can be overcome in order to obtain greater flexibility and improve other processing factors [2]. To date, primarily co-polymerization of P(3HB) with 3-hydroxyvalerate (3HV) has been investigated to increase the flexibility of polymer chains [2]. Other PHAs are under development to achieve tunable mechanical properties, such as the poly(3-hydroxyoctanoate-co-3-hydroxydecanoate) P(3HO-*co*-3HD [7]. In tissue engineering, micro/nano fibrous scaffolds have shown the ability to mimic the fibrillar part of the natural extracellular matrix (ECM), which plays a key role in cell migration, adhesion, and colonization [1]. To this purpose, electrospinning is very convenient if compared to other techniques such as spin coating, being a one-step process, providing parameter control to obtain the desired fiber morphology and providing an easy recoverability of the end product [8,9]. Specifically, electrospun fibers have an extremely high surface area to volume ratio, and the produced nonwovens show high porosity, which enable more effective cell attachment and colonization. PHA production on an industrial scale only holds a small proportion of the overall polymer market due to typically higher costs of production, but it holds the potential to reduce waste, decrease emissions, and develop green jobs [10]. Electrospinning could also promote the upscaling and industrialization of PHAs—in particular, in the biomedical sector. Versatility in the final product obtained via electrospinning allows fiber, particle, and fibril production [11]. Electrospray is typically performed via an electrospinning system using a low polymer concentration in the solution, along with other variations in parameters, in order to generate particles and fibrils often in micro or nanometric ranges [11]. The surface functionalization materials are possible by using the electrospray method.

Chitin is a natural polysaccharide found in the shells of crustaceans, cuticles of insects, and cell walls of fungi. It is the second most abundant polymerized carbon found in nature. When fibrillated on the nanoscale, chitin loses its pro-inflammatory and allergenic character and is able to proficiently interact with many cellular compounds in biological tissues [12]. Due to its positive charge, chitin nanofibrils (CN) interacts with negatively charged nanolignin (NL), derived from lignin found in plant biomass, to generate micro-complexes. Such complexes have demonstrated the ability to incorporate biomolecules such as the glycyrrhetinic acid (GA), which is derived from the licorice plant and possesses anti-inflammatory and antimicrobial properties that are interesting for treating skin disorders [13]. CN-NL/GA (CLA) complexes were developed by spray dry technology in previous studies and showed promise for skin contact applications, as CN-NL provides a suitable carrier to promote GA delivery [14,15].

The aim of this study was to set up an electrospray method to decorate the PHA surface with CN and CLA, thus providing a functionalization method for electrospun fiber meshes to be used for wound healing applications. In particular, we used a blend of P(3HB) and P(3HO-*co*-3HD) to obtain a suitable fiber morphology. CN and CLA were suspended in water-based solutions and preliminarily electrosprayed on the surface of P(3HB)/P(3HO-*co*-3HD) films to assess the best uniform nano/microparticle distribution on their surface. Thereafter, CN and CLA were electrosprayed upon P(3HB)/P(3HO-*co*-3HD) electrospun fiber meshes. Finally, we assessed which of the two chitin-based compounds had the highest beneficial effects for the epidermis, in vitro, using a human keratinocyte HaCaT cell line. The expression of a panel of cytokines involved in inflammation and immune response was investigated, including the pro-inflammatory interleukins (ILs) IL-1, IL-6, IL-8, the tumor necrosis factor α (TNF-α), the transforming growth factor β (TGF-β), and the human beta defensin 2 (HBD-2), the latter being an endogenous antimicrobial peptide. The successful fabrication of innovative surface-functionalized PHA fibers would enable the development of novel bio-based products for potential use for skin-related applications, such as wound healing or skin contact.

## 2. Materials and Methods

### 2.1. Materials

CN, NL, and GA were supplied by Mavi Sud, Aprilia (LT, Italy). Chloroform (code: 102442), 2-butanol (code: 109630), lithium bromide (LiBr_2_), acetic acid (code: 33209), ethanol (EtOH), poly(ethylene glycol) (PEG8000), and Dulbecco’s phosphate-buffered saline (DPBS) were provided by Sigma-Aldrich (Milan, Italy). Immortalized human keratinocytes, HaCaT cell line, were obtained from ATCC-LGC Standards (Milan, Italy). MgCl_2_, Dulbecco’s Modified Essential Medium (DMEM), L-glutamine, penicillin, streptomycin and fetal calf serum were purchased from Invitrogen, (Carlsbad, CA, USA). Alamar Blue was bought from Thermo Fisher Scientific (Waltham, MA, USA). LC Fast Start DNA Master SYBR Green kit was obtained from Roche Applied Science (Euroclone S.p.A., Pero, Italy).

### 2.2. P(3HO-co-3HD) and P(3HB) Production

P(3HO-*co*-3HD) was produced using *Pseudomonas mendocina* CH50 using 20 g/L of glucose in 15 L bioreactors, with 10 L working volume (Applikon Biotechnology, Tewkesbury, UK). Batch fermentation was carried out in two stages as described in Basnett et al., 2020 [7]. P(3HB) was produced using *Bacillus subtilis* OK2 using glucose at 20 g/L, using the same protocol as P(3HO-*co*-3HD) production but using a single stage batch fermentation. For simplicity P(3HB)/P(3HO-*co*-3HD) will be further referred to as PHB/PHOHD.

### 2.3. Preparation of CN and CLA Solutions and Electrospraying Protocols

CN was used at 0.52 w% in aqueous acetic acid and distilled water (50:50 *w*/*w*). The solution magnetically stirred for 3 h until it appeared uniform. The solution was electrosprayed using an electrospinning bench apparatus (Linari Engineering s.r.l., Pisa, Italy) for 20 min with a static aluminum collector with a ground charge 10 cm from the needle tip. The flow rate of 0.298 mL/h and a voltage of 15 kV were employed. CLA complexes were prepared in powder using a Buchi Mini B-190 spray drier (Flawil, Switzerland) [16] and by adding 2% CN-NL *w*/*w*% with respect to PEG. CLA complexes were thus prepared in accordance with previous works [17]. The ratio between CN and NL is 2:1 by weight. The content of GA in CLA is 0.2% by weight. CLA powder was dissolved at 0.52 *w*/*w*% in distilled water and magnetically stirred for 1 h. The solution was electrosprayed using an electrospinning bench apparatus (Linari Engineering s.r.l.) with a distance of 10 cm from the positively charged needle tip to the grounded aluminum static collector at 15 kV at a flow rate of 0.298 mL/h for 20 min.

### 2.4. PHB/PHOHD Solution Preparation

A solution of 11 *w*/*w*% PHB/PHOHD (1:10) was produced using chloroform and 2-butanol as solvents with a ratio of 70:30 (*v*/*v*). This was performed using a stepwise procedure consisting of adding PHOHD to chloroform, under magnetic stirrer for 1 h before the addition of P(3HB) and 2-butanol. The solution was left overnight under stirring to reach homogeneity before adding 0.002 g/mL LiBr_2_ and then magnetically stirred until it reached a uniform appearance.

### 2.5. Production of PHB/PHOHD Films and Fiber Meshes

The PHB/PHOHD solution at 11% (the weight of the polymers with respect to the weight of solvent mix) was poured onto a sterile glass petri dish and left for 48 h under the chemical laminar flow hood to allow full solvent evaporation. The electrospinning parameters used to produce PHB/PHOHD fiber meshes were 40 kV, a 0.5 mL/h flow rate, and a 40 cm distance from needle tip to the static aluminum collector. The process ran for 1 h. Humidity around 40% and a temperature of 20 °C was maintained throughout.

### 2.6. Electrospray of CN and CLA on PHB/PHOHD Film and Fiber Mesh

CLA and CN were electrosprayed onto the previously produced PHB/PHOHD fiber mesh and film using a flow rate of 0.298 mL/h, the voltage of 15 kV with a distance of 10 cm between the positive needle tip and grounded static collector for 60 min.

### 2.7. Morphological Characterization

Morphological analysis of the samples was performed using field emission electron scanning microscopy (FE-SEM) with FEI FEG-Quanta 450 instrument (Field Electron and Ion Company, Hillsboro, OR, USA) and Inverted optical microscope (Nikon Ti, Nikon Instruments, Amsterdam, The Netherlands). The samples were sputtered with gold or platinum for analysis. Image J software (version 1.52t) was used to evaluate the size of nanofibrils and fibers. The average of 50 measurements has been reported for each sample.

### 2.8. Chemical Structure Characterization

Infrared spectroscopy using Nicolet T380 instrument (Thermo Scientific, Waltham, MA, USA) equipped with a Smart ITX ATR attachment with a diamond plate was employed for chemical structure characterization of both solid chitin-based substances and electrospun/electrosprayed samples.

### 2.9. Evaluation of HaCaT Cell Line Viability

Each material sample was sterilized overnight in absolute ethanol, then rinsed three times with PBS. HaCaT cells were cultured in D-MEM supplemented with 1% Penicillin-streptomycin, 1% L-glutamine and 10% fetal calf serum in a humidified incubator set at 37 °C in 95% air and 5% CO_2._ The materials were put in contact with HaCaT cells seeded in 12-well plates for 6 h and 24 h. At the endpoint, the Alamar blue test was performed following the manufacturer’s protocol after 4 h incubation with the dye. Briefly, Alamar blue incorporates a redox indicator that changes color according to cell metabolic activity. The supernatants were read with a spectrophotometer using a double wavelength reading at 570 nm and 600 nm. Finally, the reduced percentage of the dye (%AB_RED_) was calculated by correlating the absorbance values and the molar extinction coefficients of the dye at the selected wavelengths.

### 2.10. Anti-Inflammatory and Immune Responses Evaluation of HaCaT Cells

The immunomodulatory properties of PHB/PHOHD fiber meshes electrospun with CN and CLA were assayed using HaCaT cells. The cells, cultured as described above, were seeded inside 12-well plates until 80% of confluence was reached. At the time-points of the experiment (6 h and 24 h), the mRNA was extracted from the cells and the levels of expression of the proinflammatory cytokines IL-8, IL-6, IL-1β, IL-1 α, and TNF-α anti-inflammatory cytokine TGF-β and antimicrobial peptide HBD-2 were evaluated by real-time reverse transcriptase polymer chain reaction (RT-PCR). Briefly, the total RNA was isolated with TRizol, and 1 µm of RNA was reverse-transcribed into complementary DNA (cDNA) using random hexamer primers at 42 °C for 45 min, according to the manufacturer’s instructions. PCR was carried out with the LC Fast Start DNA Master SYBR Green kit using 2 µL of cDNA, corresponding to 10 ng of total RNA in a 20 µL final volume, 3 mM MgCl_2_, and 0.5 µM sense and antisense primers (Table 1). The results were normalized by the expression of the same cytokine in untreated cells, as a control.

## 3. Results

### 3.1. Morphological Characterization

#### 3.1.1. Morphological Characterization of Electrosprayed CN and CLA

**Figure 1:**
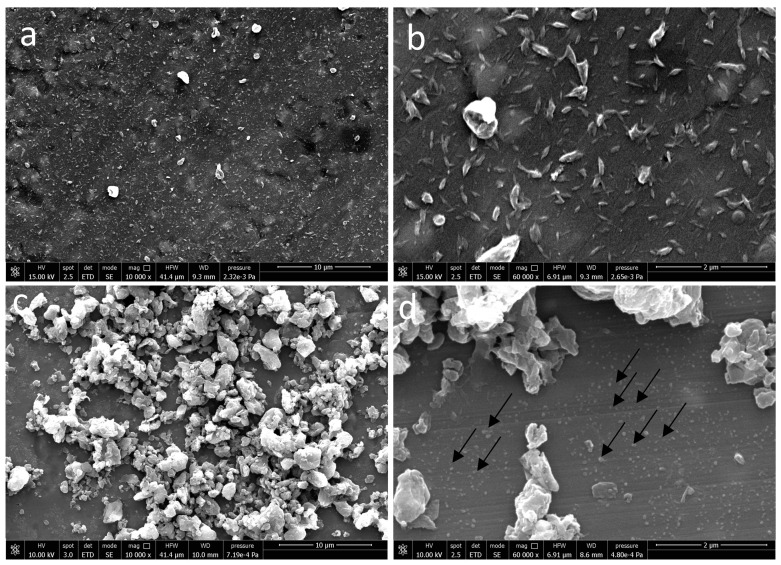
Scanning electron microscopy (SEM) micrographs of (**a**,**b**) electrosprayed CNs at 10,000× and 60,000× magnifications and (**c**,**d**) electrosprayed CN -NL assembled into micro-complexes loaded with (GA), i.e., (CLA) complexes, at 10,000× and 60,000× magnification. Arrows show subpopulation of CLA complexes with low size.

On aluminum foil as a substrate, electrosprayed CN suspensions allowed us to obtain uniform surface decoration (Figure 1a).

CNs were well dispersed and showed an average size of 180 nm ± 47 nm (Figure 1b). Two main subpopulations of CLA complexes with an average size of 65 ± 20 nm (shown with arrows) and 1239 ± 626 nm were observed (Figure 1c,d). There are also some aggregations on CLA fibrils.

#### 3.1.2. Morphological Analysis of Functionalized PHB/PHOHD Films

**Figure 2:**
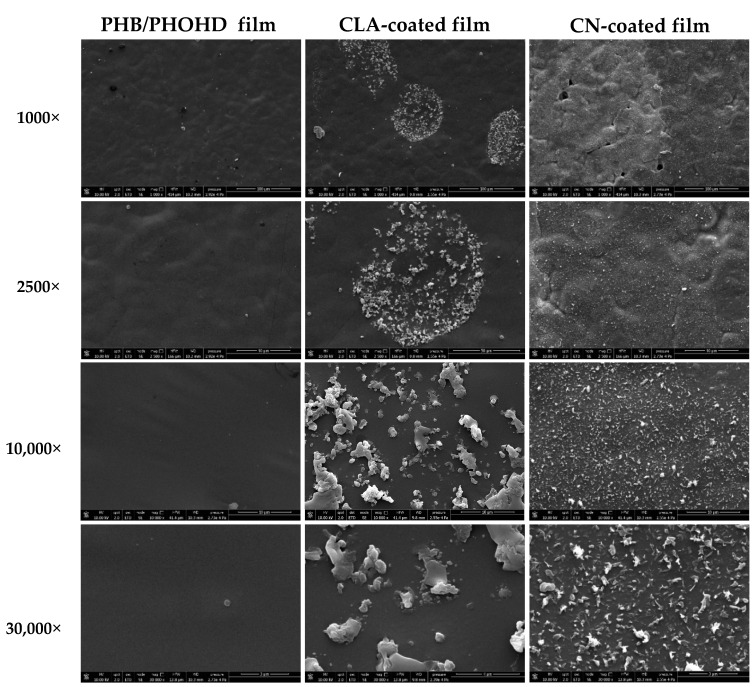
SEM micrographs of PHB/PHOHD films, PHB/PHOHD films functionalized with CNs, and PHB/PHOHD films functionalized with CLA complexes, observed at different magnifications.

Figure 2 shows SEM images of the surface of PHB/PHOHD films plain and functionalized with CNs or CLA complexes at different magnifications. CNs with uniform size and morphology were homogeneously electrosprayed and coated the whole surface of PHB/PHOHD films. CLA complexes distributed locally, and thus non-uniformly, on the surface of the films. The aggregated CLA microparticles can also be observed on the surface of the films.

#### 3.1.3. Morphological Analysis of Functionalized PHB/PHOHD-Electrospun Fiber Meshes

**Figure 3:**
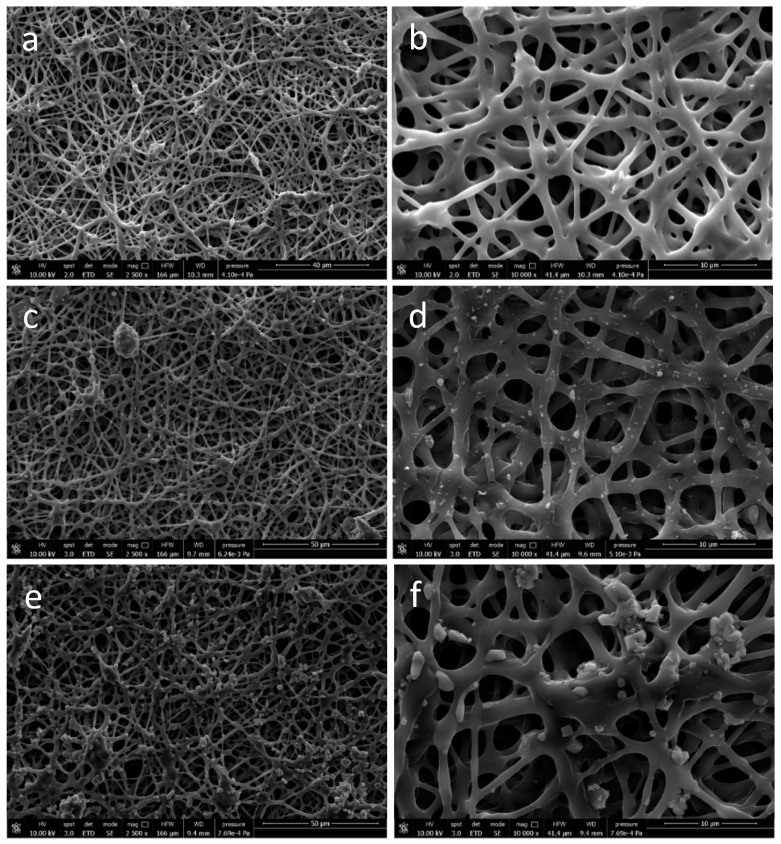
SEM images of (**a**,**b**) PHB/PHOHD electrospun fiber meshes at different magnifications: (**a**) 2500× (**b**) 10,000×; (**c**,**d**) PHB/PHOHD electrospun fiber meshes functionalized with electrosprayed CN at different magnifications: (**c**) 2500×, (**d**) 10,000×; (**e**,**f**) PHB/PHOHD electrospun fiber meshes functionalized with electrosprayed CLA at different magnifications—(**e**) 2500×, (**f**) 10,000×.

**Figure 4:**
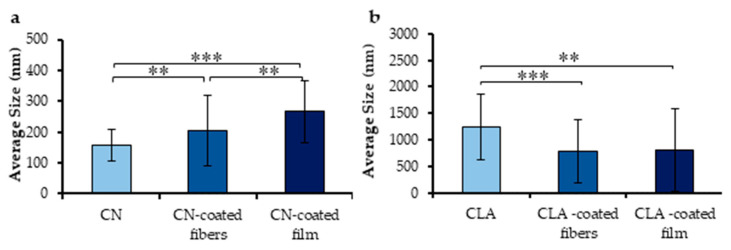
Size of electrosprayed CNs (**a**) and CLA complexes (**b**) on different surfaces—no polymer (aluminum foil), PHB/PHOHD fibers, and PHB/PHOHD film. Data are expressed as ± SD (*n* = 50 particles), ** *p* < 0.001; *** *p* < 0.0001.

The electrospun meshes displayed anisotropic fibrous morphology with a homogenous fiber feature (Figure 3a). Ultrafine PHB/PHOHD fibers with an average diameter of 1.28 ± 0.58 µm were successfully produced (Figure 3b). By using PHB/PHOHD fibers as a substrate, CNs (Figure 3c,d) and CLA (Figure 3e,f) were uniformly electrosprayed on the surface of fibers. Figure 4 shows the size distribution of electrosprayed CNs and CLA complexes on the surface of PHB/PHOHD fibers and films. After electrospraying, CNs were found with lower size on the surface of the fibers than on films, although still more aggregated than on the aluminum foil (*p* < 0.05) (Figure 4a). In an opposite manner, CLA microparticles exhibited lower size and size distribution on the surface of fibers and film than on the aluminum foil (Figure 4b).

### 3.2. Chemical Characterization

#### 3.2.1. Chemical Characterization of (PHB/PHOHD)-Electrospun Fiber Mesh and Film

**Figure 5:**
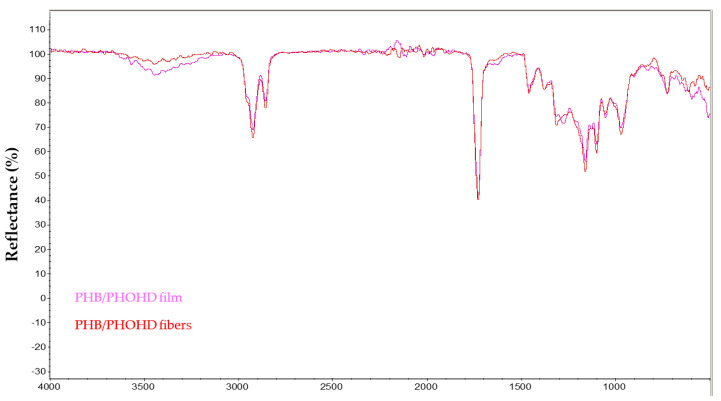
Fourier-transform infrared spectroscopy (FTIR) spectra of PHB/PHOHD film and (PHB/PHOHD)-electrospun fibers.

The Fourier-transform infrared spectroscopy (FTIR) spectra of PHB/PHOHD-electrospun fibers and film were compared (Figure 5) by normalizing on the peak at 1730 cm^−1^, attributable to the C=O stretching of the ester group of PHA.

Weaker peaks at 3500 cm^−1^ and 1600 cm^−1^ in the fiber mesh indicated a reduction in O-H stretching. As this band intensity highly depends on the humidity content of the samples, that can be influenced by ambient conditions, this change cannot be attributed to a change in the –OH concentration of the samples but more reasonably to some water content fluctuations. Overall, there is no strong indication of any detrimental effect on the polymer structural integrity during the electrospinning process.

#### 3.2.2. Chemical Characterization of CN-Coated PHB/PHOHD Fibers

**Figure 6:**
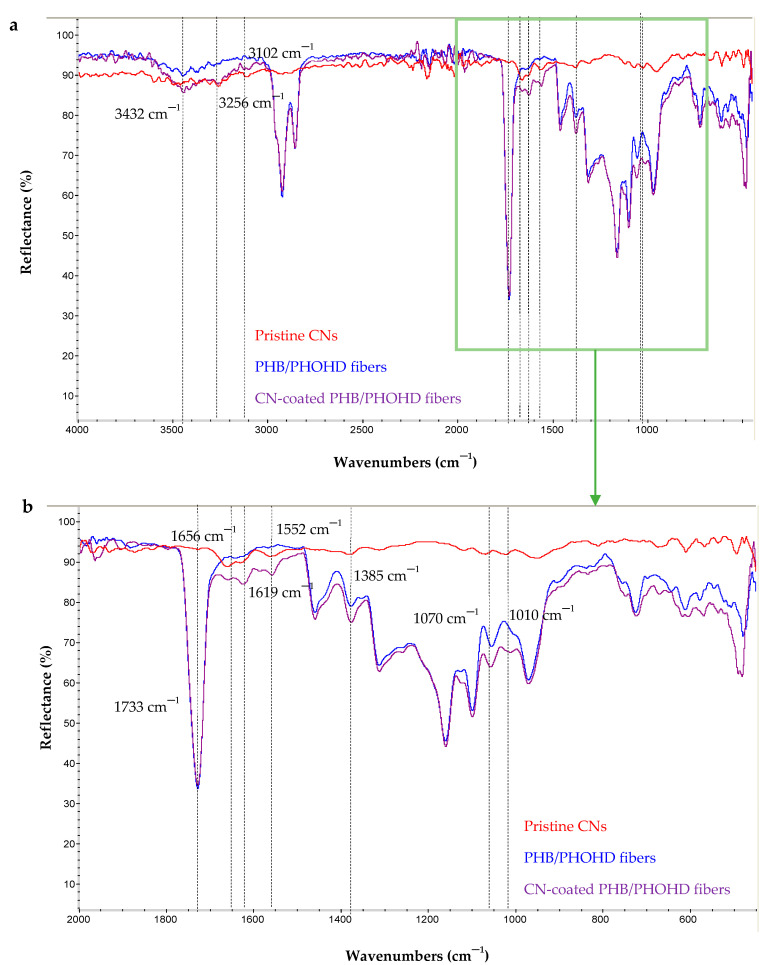
FTIR spectra of pristine CN, PHB/PHOHD-electrospun fibers, both CN-coated and uncoated (plain). (**a**) The whole investigated spectrum and (**b**) Zoomed-in spectrum in the 2000–1100 cm^−1^ range.

The dried pristine CNs, PHB/PHOHD fibers and CN-coated PHB/PHOHD fibers were characterized by FTIR (Figure 6). The characteristic bands of CN are 1010 cm^−1^ and 1070 cm^−1^, typical of C–O stretching, 1552 cm^−1^ attributed to amide II, 1619 cm^−1^ and 1656 cm^−1^ attributed to amide I, 2874 cm^−1^ attributed to C–H stretching, 3102 cm^−1^ and 3256 cm^−1^ attributed to N–H stretching of the amide and amine groups, and 3439 cm^−1^ attributable to O–H stretching. All these bands were observed in the FTIR spectrum of CNs.

The main characteristic bands of CN (1552 cm^−1^, 1619 cm^−1^ and 1656 cm^−1^) can be also observed on CN-coated PHB/PHOHD fiber spectrum. The increased intensity of the band at 1010 cm^−1^, 1070 cm^−1^, and 1385 cm^−1^ was clearly detected in the spectrum of CN-coated fibers. Indeed, the ratio of bands at 1010 cm^−1^ and 1070 cm^−1^, attributable to C–O linkages (abundant in polysaccharides like CN), to the reference band of 1733 cm^−1^ was higher for the CN-coated PHB/PHOHD fibers than for the plain PHB/PHOHD fibers.

These observations corroborated the presence of CNs on the surface of PHB/PHOHD fibers.

#### 3.2.3. Chemical Characterization of CLA-Coated PHB/PHOHD Fibres

**Figure 7:**
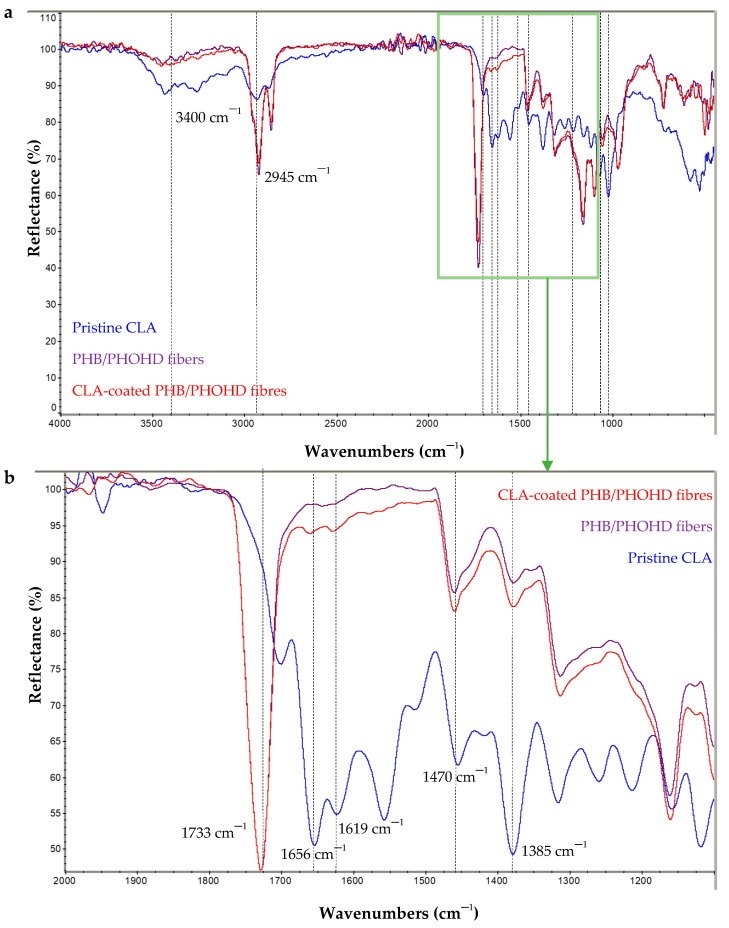
FTIR spectra of CLA complexes, PHB/PHOHD-electrospun fibers both CLA-coated and uncoated (plain). (**a**) The whole investigated spectrum and (**b**) zoomed-in spectrum in 2000–1100 cm^−1^.

CLA complexes, PHB/PHOHD fibers and CLA-coated PHB/PHOHD fibers were characterized by FTIR (Figure 7). The characteristic bands of CN described above, and NL, namely, 1052 cm^−1^ attributed to aromatic C–H deformation [18]; 1222 cm^−1^ associated with C–C plus C–O, 1511 cm^−1^ and 1601 cm^−1^ associated with aromatic skeleton vibrations; and GA, namely 3430 cm^−1^ typical of OH stretching and 2945 cm^−1^ attributable to CH stretching; 1700 cm^−1^ and 1660 cm^−1^ attributed to the C=O stretching of carboxylic and ketone groups, respectively, and 1470 cm^−1^ to CH_2_ bending, 1025 cm^−1^ to C–O stretching, and 990 cm^−1^ attributed to the rocking of the methyl group, were detected in the CLA complex spectra. The main characteristic bands of CLA complexes were considered, 1385 cm^−1^, 1470 cm^−1^, 1619 cm^−1^, and 1656 cm^−1^, which were present in the FTIR spectra of functionalized PHB/PHOHD fibers. In addition, a higher ratio of CLA characteristic bands with respect to 1733 cm^−1^, as a reference band, were obtained for the coated fibers than uncoated ones, which demonstrated an effective functionalization.

### 3.3. HaCaT Cell Metabolic Activity

AlamarBlue^®^ test was performed on the HaCaT cells in order to assess the cytocompatibility of the CN-coated fibers and CLA-coated fiber meshes. The results obtained highlighted good cytocompatibility, even though the low concentration of the CN-based particles reduced the metabolic activity as compared to that of the plain polymer (Table 2).

### 3.4. Immunomodulatory Properties

**Figure 8:**
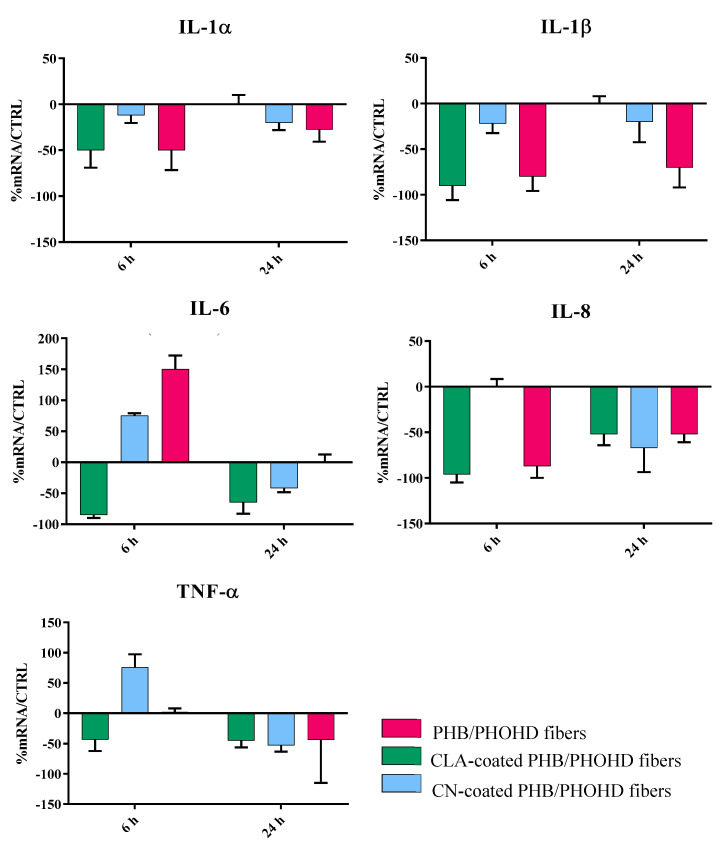
Bar graphs showing the results of real time RT-PCR performed related to different cytokines involved in the inflammatory response of HaCaT cells after being exposed to the PHB/PHOHD fibers (plain, CLA-coated, and CN-coated) for 6 h and 24 h. The results were normalized by the expression in untreated cells as control.

The results show that the samples were able to induce a powerful anti-inflammatory activity in HaCaT cells (Figure 8).

**Figure 9:**
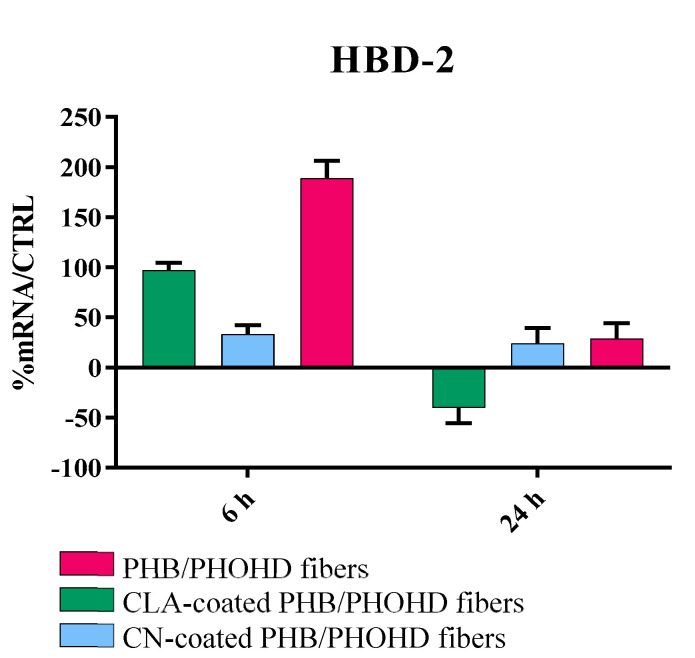
Bar graph showing the results of real time RT-PCR analysis for HBD-2 produced by HaCaT cells exposed to the PHB/PHOHD fibers (plain, CLA-coated, and CN-coated) at 6 h and 24 h. The results are normalized by the expression in untreated cells as control.

Indeed, all the samples strongly downregulated the expression of the main proinflammatory cytokines IL-1 (α and β), IL-6, IL-8 and TNF-α after 24 h, and most of them within 6 h. The plain fibers and the fibers coated with CNs initially upregulated IL-6; however, this cytokine was subsequently downregulated. TGF-β was not modulated with respect to the untreated cells. In addition, the samples showed the ability to induce the eαpression of HBD-2 in HaCat cells (Figure 9) after 6 h exposure, thus suggesting a role in stimulating an indirect antibacterial activity.

## 4. Discussion

The skin is the first organ to be injured, since it protects the other tissues and organs of the body, so it retains a high capacity for self-repairing. However, the natural wound-healing process of skin may be ineffective in cases of profound lesions or large surface loss, leading to detrimental and painful conditions that require repair adjuvants or tissue substitutes. For example, wounds deriving from diabetes show difficultly when healing and tend to become chronic via self-inflammation. Such a process causes the degradation of the growth factors deputed to healing, thus concurring to generate pathologic states, like recurrent infections and tumor onset. Therefore, the application of specific biomaterial dressings able to stimulate and/or accelerate the wound healing process can be of great importance to restore the normal native tissues [19]. In a lifetime, many other events also induce skin damage and irritation; therefore, anti-inflammatory biomaterials find a number of applications in skin contact products.

In our study, we produced electrospun PHA fibers with improved antibacterial and anti-inflammatory properties as a bioactive nonwoven able to promote skin self-repair and prevent the infection processes. PHAs are highly biocompatible and naturally occurring bacteria-derived polyesters and are produced by bacterial fermentation [20,21]. In particular, we used a blend of two PHAs (i.e., P(3HB) and P(3HO-*co*-3HD)), which allowed the best fiber production process. The P(3HB)/P(3HO-*co*-3HD) blend was used in order to achieve a good balance between the good processability of P(3HB) and the desirable elastomeric property of P(3HO-*co*-3HD). Different techniques such as solvent casting, dip molding, and 3D printing have been used for processing of PHAs in different structures for a variety of biomedical applications [22,23]. Fibrous PHA scaffolds with specific characteristics can promote tissue formation, since their topography is similar to the fibers and fibrils naturally found in the tissue extracellular matrix (ECM), and can be produced by electrospinning as a versatile method [24,25,26]. Salvatore et al. demonstrated the potential of electrospun PHB/collagen meshes as suitable substrates for wound healing, with the PHB/collagen ratio controlling the morphological, mechanical, and degradation properties of the meshes [27].

The formation of beads during the electrospinning process is a common phenomenon that can affect the quality of the fibers [4]. By using appropriate electrospinning conditions, including solvent mixture and LiBr_2_, we produced beadless PHB/PHOHD ultrafine fibers with a homogeneous morphology and quite uniform diameter. We showed that electrospinning is a safe technology to produce the ultrafine fibers since no significant difference was observed in the chemical structure of electrospun fibers in comparison to solvent-casted films.

CNs and CLA complexes are bio-based nano- and micro-compounds that can be considered useful bioactive agents for functionalizing skin contact substrates, as they showed proficient interaction in an in vitro skin model [14]. It is important to obtain an easy and effective method for applying such components to biomaterial surfaces to put in contact with skin, encompassing bulk incorporation and surface functionalization [15,28]. In fact, if plain CNs are applied as water suspension, further steps like evaporation must be taken into account. Moreover, the substrate surface may be affected by wetting. On the other hand, when CNs are used to incorporate bioactive molecules, such as GA in CLA complexes, it is important to avoid as much as possible any processes that would lead to possible dispersion or modification of the bioactive molecule. We considered that electrospraying could be a valuable method for CN and CLA complex deposition on PHA surfaces, as it can be performed with the same equipment used for fiber production without any post treatment. At first, PHB/PHOHD films were used to set up electrospraying conditions. Indeed, the chemical nature of the substrate used as a collector membrane can affect the local electric field and cause different deposition outcomes [29]. We observed a different behavior of the CN and CLA solutions electrosprayed on PHB/PHOHD films. Specifically, CNs were even, whereas CLA complexes were locally dispersed. This is a consequence of the diverse interaction of positively charged CNs and the neutral CLA complexes with the electric field. Subsequently, the same technique was used to decorate the surface of the electrospun P(3HB)/P(3HO-*co*-3HD) fibers in order to improve their antibacterial and anti-inflammatory properties. Due to the porous nature of these nonwovens, both CNs and CLA complexes were homogeneously delivered to the fiber surface, possibly due to a more powerful interaction of the metallic collector with the particles. SEM analysis demonstrated the presence of both CNs and CLA complexes on the surface of P(3HB)/P(3HO-*co*-3HD) fibers and films. Using different substrates also led to the formation of particles with different size caused by aggregation. This polydispersion effect can be attributed to the interaction of different substrates with the electric field applied during electrospray too [30,31]. However, in all the tested substrates, electrosprayed CN had smaller and more uniform size than electrosprayed CLA. FTIR results also confirmed the presence of CN and CLA nanofibrils on the surface of fibers without any remarkable change in their chemical structures. These results demonstrate that electrospray is a suitable technique for functionalizing the substrates for wound healing applications.

Since the nanosized materials can migrate into tissues and potentially induce unknown reactions, studying the interaction with skin cells is fundamental to demonstrate safety in skin contact applications [25,31,32]. In this study, bio-based and bioresorbable materials were selected to minimize the harmful risks due to organ accumulation, while providing beneficial bioactive properties. The short term interaction of the functionalized substrates with human keratinocytes was investigated in vitro using HaCaT cells. Preliminary investigation of cell metabolic activity suggested good cytocompatibility, as also observed in previous studies [14]. In order to predict the reaction of the epidermis layer, the expression of an array of cytokines involved in the inflammation response were studied, including IL-1, IL-6, IL-8, and TNF-α [14]. Cytokines are multi-functional biological molecules that are involved in autocrine, paracrine, and endocrine signaling as immunomodulating agents and play an important role in biological activities such as tissue repair, growth, and cell development [33]. In particular, IL-1 promotes local inflammation and coagulation, increases the expression of adhesion molecules, and causes the release of chemokines and recruitment of leukocytes to the site of inflammation [34]. IL-6 is involved in the inflammatory acute phase response. IL-8 is a multi-functional chemokine, inducing the activation of polymorphonuclear leukocytes and angiogenesis [35]. Finally, TNF-α is an essential mediator in inflammation with reactive roles in blood coagulation process [36]. All these cytokines were strongly downregulated in all the produced samples, showing that these nano- and micro-compounds in combination with the P(3HB)/P(3HO-*co*-3HD) fibers inherently support the reduction of inflammatory states without the upregulation of TGF-β.

The PHB/PHOHD fiber meshes, plain or functionalized with CNs and CLA complexes, were also able to upregulate the HaCaT cell expression of an antimicrobial peptide, HBD-2, which acts as an endogenous antibiotic and plays an important role in the innate immune response [37]. Indeed, if the healing process does not proceed properly, a wound may become chronic, thus it is not able to self-repair in a normal, orderly, and timely manner and is highly prone to infections. Since traditional treatment methods are not sufficient for changing the microenvironment of chronic wounds, they do not represent breakthrough approaches [38]. Recently, Zhang et al. comprehensively reviewed the functional biomaterials that can improve chronic wound healing through debridement, anti-infection and antioxidant effects, immunoregulation, angiogenesis, and ECM remodeling [39]. They pointed out that functional biomaterials are expected to improve the patients’ quality of life via resolving the treatment dilemma for chronic wounds. Functional biomaterials are also expected to elicit an appropriate immune response, thus orchestrating a cascade of biological events that promote safe and uncomplicated healing. To this purpose, it is important to design scaffold topography that helps both dermal cell organization and epidermal cell migration. Even though many scaffold architectures have been revealed to support fibroblast growth [40], by mimicking the structure of the fibrillar ECM, electrospun scaffolds are expected to allow an optimal dermal regeneration [26]. Moreover, by downregulating inflammatory cytokines and upregulating HBD-2 in keratinocytes, thanks to CN-based particles, such surface functionalized scaffolds should act as highly functional biomaterials [41].

In this view, the development of bio-based and biodegradable functional nonwovens with powerful anti-inflammatory activity and indirect antimicrobial properties opens an interesting scenario in skin repair and regeneration products, including, but not limited to, wound dressings, which can use a sustainable and green route.

## 5. Conclusions

We developed an easy and effective method to obtain surface-decorated electrospun nonwovens via electrospray of CN and CLA complex solution of P(3HB)/P(3HO-*co*-3HD) fiber meshes. Such completely bio-based and biodegradable functional nonwovens possessed strong anti-inflammatory activity as they downregulated the main pro-inflammatory cytokines and exhibited the capacity of stimulating HBD-2 by human keratinocytes. Having green and effective substrates for skin contact and repair would allow better treatment of irritated skin and complex skin wounds.

## Figures and Tables

**Table 1:** Real-time reverse transcriptase polymer chain reaction (RT-PCR) details, including gene, primer sequences, operational conditions, and product size.

Gene	Primers Sequence	Conditions	Product Size (bp)
IL-1α	5′-CATGTCAAATTTCACTGCTTCATCC-3′	5 s at 95 °C, 8 s at 55 °C, 17 s at 72 °C for 45 cycles	421
	5′-GTCTCTGAATCAGAAATCCTTCTATC-3′		
IL-1β	5′-GCATCCAGCTACGAATCTCC-3′	5 s at 95 °C, 14 s at 58 °C, 28 s at 72 °C for 40 cycles	708
	5′-CCACATTCAGCACAGGACTC-3′		
IL-6	5′-ATGAACTCCTTCTCCACAAGCGC-3′	5 s at 95 °C, 13 s at 56 °C, 25 at 72 °C for 40 cycles	628
	5′-GAAGAGCCCTCAGGCTGGACTG-3′		
IL-8	5′-ATGACTTCCAAGCTGGCCGTG-3′	5 s at 94 °C, 6 s at 55 °C, 12 s at 72 °C for 40 cycles	297
	5′-TGAATTCTCAGCCCTCTTCAAAAACTTCTC-3′		
TNF-α	5′-CAGAGGGAAGAGTTCCCCAG-3′	5 s at 95 °C, 6 s at 57 °C, 13 s at 72 °C for 40 cycles	324
	5′-CCTTGGTCTGGTAGGAGACG-3′		
TGF-β	5′-CCGACTACTACGCCAAGGAGGTCAC-3′	5 s at 94 °C, 9 s at 60 °C, 18 s at 72 °C for 40 cycles	439
	5′-AGGCCGGTTCATGCCATGAATGGTG-3′		
HBD-2	5′-GGATCCATGGGTATAGGCGATCCTGTTA-3′	5 s at 94 °C, 6 s at 63 °C, 10 s at 72 °C for 50 cycles	198
	5′-AAGCTTCTCTGATGAGGGAGCCCTTTCT-3′		

**Table 2:** AlamarBlue results on PHB/PHOHD fiber meshes, coated with CNs or CLA complexes.

Sample	%AB_RED_
PHB/PHOHD fiber mesh	76
CLA-coated PHB/PHOHD fiber mesh	64
CN-coated PHB/PHOHD fiber mesh	69

## Abbreviations

CLA	Chitin nanofibril/nanolignin-glycyrrhetinic acid
CN	Chitin nanofibril
DMEM	Dulbecco’s modified essential medium
DPBS	Dulbecco’s phosphate-buffered saline
EtOH	Ethanol
GA	Glycyrrhizin acid
HBD-2	Human beta-defensin 2
IL	Interleukin
NL	Nanolignin
P(3HB)	Poly(3-hydroxybutyrate)
P(3HO-*co*-3HD)	Poly(3-h ydroxyoctanoate-*co*-3-hydroxydecanoate)
PEG	Poly(ethylene glycol)
PHAs	Polyhydroxyalkanoates
TGF-β	Transforming growth factor beta
TNF-α	Tumor necrosis factor alpha
